# Population centroids of the world administrative units from nighttime lights 1992-2013

**DOI:** 10.1038/s41597-019-0250-z

**Published:** 2019-10-28

**Authors:** Ola Hall, Maria Francisca Archila Bustos, Niklas Boke Olén, Thomas Niedomysl

**Affiliations:** 1 Department of Human and Economic Geography, Lund University, Sölvegatan 10, S-223 00 Lund, Sweden; 2 Centre for Environmental and Climate Research, Lund University, Sölvegatan 37, S-223 62 Lund, Sweden; 3 Department of Analysis, Region Halland, Södra vägen 9, P.O. Box 517, S-301 80 Halmstad, Sweden

**Keywords:** Geography, Society

## Abstract

Knowledge about the past, current and future distribution of the human population is fundamental for tackling many global challenges. Censuses are used to collect information about population within a specified spatial unit. The spatial units are usually arbitrarily defined and their numbers, size and shape tend to change over time. These issues make comparisons between areas and countries difficult. We have in related work proposed that the shape of the lit area derived from nighttime lights, weighted by its intensity can be used to analyse characteristics of the population distribution, such as the mean centre of population. We have processed global nighttime lights data for the period 1992–2013 and derived centroids for administrative levels 0–2 of the Database of Global Administrative Areas, corresponding to nations and two levels of sub-divisions, that can be used to analyse patterns of global or local population changes. The consistency of the produced dataset was investigated and distance between true population centres and derived centres are compared using Swedish census data as a benchmark.

## Abstract (summary)

**Table:** 

Measurement(s)	light • Population Density
Technology Type(s)	digital curation • computational modeling technique
Sample Characteristic - Organism	Homo sapiens
Sample Characteristic - Location	Earth (planet)

Machine-accessible metadata file describing the reported data: 10.6084/m9.figshare.9939494

## Background & Summary

Uneven population distribution and population change trends have important effects on society and landscapes. Knowledge about the past, current and future distribution of the human population is fundamental for tackling many global challenges such as food security, climate change adaptation, and poverty reduction. Detailed data on human population distribution is therefore vital. However, such data is most often aggregated to administrative areas, as is the case with census data, and in some cases may even be non-existent.

Censuses constitute the typical way of collecting information about a population within a tract or any spatial unit. Commonly the unit is the nation with all its subdivisions. From an analytical perspective the typical data collection framework used to record population is problematic. The spatial units, typically administrative, used by each nation are usually arbitrarily defined and their numbers, size and shape tend to change over time^1^. The aggregated nature of census data means that an assumption is made about the equal distribution of people within administrative units. Carrying out a census is also a costly and cumbersome undertaking. The revisit time is at best once a year but more commonly once every 5–10 years. In addition, accurate census estimates can be limited by inability to count inaccessible populations, whether this is for political, economic or even environmental reasons.

In recent years, nighttime lights imagery has emerged as a popular proxy for human activity and has been used to simulate human population distribution^2^ and even to generate detailed population estimates at the pixel level^3–5^. Such data has the potential to address some of the previously discussed limitations of census data due to its high spatial and temporal resolution and disaggregated nature. However, what is required is a methodology for simulating human population distribution using this nighttime lights data.

We have, in previous work, shown that the centroid of any population can be located with high accuracy without knowing the actual population distribution through the use of nighttime lights imagery alone^6^. We showed that the shape of the lit area, weighted by its intensity could be used to simulate characteristics of the population distribution, such as the mean centre of population (also referred to as the centroid).

In this paper we expand on our previous work to generate a global nighttime lights centroid dataset. Population centroids are valuable in many fields of social science and business where locational analysis, interactions, flows, and network modelling are used. Population centroids are in these instances much more beneficial than using geographic centroids which are located in the geographic center of administrative areas. We have, therefore, processed global nighttime lights data for the period 1992–2013 and derived annual centroids for administrative levels 0–2 of the GADM (Database of Global Administrative Areas), corresponding to nations and two levels of sub-divisions. This data is consistent in both time and space and can thus be used to analyse patterns of global or local population distribution changes without many of the typical caveats associated with traditional population data. We propose that population centroids based on nighttime lights data can be used in applications that require knowledge of human population distribution but where full time-series may not be available or where analyses span over many different countries.

One such application, which we have previously illustrated, is in migration research where estimation of migration distances has for long been hampered by inconsistent data^7^. Distance is key not only for definitional purposes but also to understand migrants’ characteristics and reasons for migration^1,8^. Migration distances are generally derived based on the area centroid of administrative tracts and the measured distance between these resulting centroids. This approach provides an estimate of migration distance but the location of the administrative centroid might, for obvious reasons, not correspond well with the location of the population within the administrative tract. Greater precision can be achieved by weighting area centroids by population^6^ and thus locating a more accurate “population centre”. However, locating the population-weighted centre requires population data at a high spatial level, which is typically not available or not accessible due to integrity reasons. The nighttime lights centroids derived here could serve as a proxy for population centroids in migration distance applications.

There are, of course, limitations to the generated centroid dataset. Some of these are related to inherent issues in the nighttime lights data. One such issue is that the nighttime lights data used is known to overestimate the lighted area on the ground which and can lead to shifts in centroids away from the true population centroid. Additionally, there is a loss of accuracy in nighttime lights data above approximately 60 to 65°N because of late sunsets and high amounts of aurora activity^9^ which can impact centroids in those latitudes. Other limitations are related to the boundary data used to generate the centroids, which are subject to the Modifiable Area Unit Problem (MAUP), meaning that, if other boundaries were used, centroids might shift. Additionally, the irregular nature of administrative boundary polygons can lead to centroids which do not fall within the corresponding administrative area. Finally, there are limitations associated with administrative areas that are composed of multiple polygons, as is the case with some island nations, for example, because the disaggregated nature of the polygons leads to centroids which may potentially be pulled away from the true population center. These limitations are important to consider depending on the study area and scale of analysis.

## Methods

The centroid datasets were created using two different algorithms for locating weighted geographic centres. The first was that described by Barmore^10^ and the second was that described by Aboufadel and Austin^11^. Before generating the centroid datasets, the input nighttime lights data were calibrated to allow for cross-year analysis. These methods are expanded versions of descriptions in our related work^6^.

Annual nighttime light intensity data from the digital data archive at the National Oceanic and Atmospheric Association’s National Geophysical Data Center (NOAA-NGDC) were used. The Version 4 DMSP-OLS Stable Lights time series consists of 34 tiff raster layers collected by six different satellites over 22 years (1992–2013). During some years there are multiple satellites collecting data from which an average value was extracted. Administrative boundaries were taken from the GADM database of Global Administrative Areas, version 2.8. A shapefile of locations of gas flares, produced by Elvidge, *et al*.^12^, was used in calibration. See Table 1 for a summary of datasets.

**Table 1:** Summary of included datasets.

Name	Description	Source	Version	Data	Spatial coverage (°)	Spatial resolution	Temporal coverage
DMSP-OLS	Nighttime lights	NOAA-NGDC^24^	v. 4, 2014	Geotiff	−180–180, −65–75	30 arc sec	1992–2013
GADM	Global administrative area boundaries	GADM database^25^	v.2.8, 2015	Polygon Shapefile	Global	N/A	N/A
Global Gas Flaring	Gas flares mask polygons	NOAA-NGDC^24^	N/A	Polygon Shapefile	Global	N/A	N/A

### Calibration and gas flare removal

The Operational Linescan System (OLS) flown by the U.S. Air Force Defence Meteorological Satellite Program (DMSP) collects nighttime lights data. The stable lights dataset is an annual average digital brightness (DN) product, processed to remove ephemeral lights, clouds and background noise. In order to allow cross-year analysis it must be calibrated against a reference year since the visible band on the OLS has no in-flight calibration and the sensors initially differ in their radiometric performance and since times of and conditions under image acquisition vary.

Here, a calibration method developed by Elvidge, *et al*.^12,13^, referred to as the invariant region and quadratic regression (IRQR) method, was used. This is a regression-based calibration method which takes a reference region where lighting intensity has changed very little over time and uses this region to standardize the datasets. Various other calibration methods have been developed and tested in the literature^14–17^. Most of these methods are based on the IRQR method and make the same assumptions; however, they are either region specific, adopt different calibration sites or use different regression methods^18^. The IRQR method remains the most widely used calibration method for its simplicity and adaptability and for this reason it was selected for this application.

Following Elvidge, *et al*.^12,13^, a reference area where lighting has remained constant throughout the study period was selected and used to develop a linear regression equation between the DN values for each individual satellite product in that area against those of a reference satellite product. The regression equation was applied to the global dataset to convert the DN values into a common range.

Satellite F18 for year 2010 (F182010) was selected as the reference satellite product because it contained the highest average DN values. Twenty candidate calibration regions were selected and scattergrams of the DN values were plotted against the reference satellite product in order to find a reference area with minimal change in lighting over time. This is defined as an area with a scattergram that has a clearly defined diagonal axis and minimal width along the primary axis^13^. Sicily was ultimately selected as the reference area. This is consistent with the findings and methods in the literature^12,13^.

Next, a second order regression was estimated for Sicily for each satellite product, as shown in Eq. 1, where y is the calibrated DN value; x is the original DN value; and C0, C1 and C2 are the calibration coefficients. The calibration coefficients where then applied globally to each satellite product. See Table 2 for coefficients used for calibrating each image. The individual satellite products were then averaged by year in order to create 22 final calibrated rasters, one for each year from 1992 to 2013.

**Table 2:** Coefficients for calibration of DN values in nighttime lights time series.

Satellite	Year	C_0_	C_1_	C_2_
F10	1992	0.1490	1.8118	−0.0150
F10	1993	0.0361	1.8989	−0.0164
F10	1994	0.1513	1.8856	−0.0163
F12	1994	0.1557	1.5294	−0.0097
F12	1995	0.0615	1.6362	−0.0117
F12	1996	0.1518	1.7035	−0.0130
F12	1997	0.0519	1.5594	−0.0102
F12	1998	0.0613	1.4546	−0.0090
F12	1999	0.1533	1.4074	−0.0088
F14	1997	0.0635	2.0794	−0.0192
F14	1998	0.2294	2.0338	−0.0196
F14	1999	0.1086	1.9149	−0.0172
F14	2000	0.1730	1.8645	−0.0163
F14	2001	0.1008	1.7736	−0.0145
F14	2002	0.1457	1.6906	−0.0130
F14	2003	0.1093	1.7570	−0.0146
F15	2000	0.0103	1.4332	−0.0085
F15	2001	0.0394	1.4447	−0.0086
F15	2002	0.0930	1.3740	−0.0078
F15	2003	0.0749	1.9672	−0.0174
F15	2004	0.1892	1.8187	−0.0153
F15	2005	0.1003	1.7477	−0.0137
F15	2006	0.1079	1.7860	−0.0143
F15	2007	0.1882	1.8613	−0.0158
F16	2004	0.0870	1.6205	−0.0114
F16	2005	0.0787	1.8225	−0.0153
F16	2006	0.1096	1.5518	−0.0102
F16	2007	0.1046	1.3652	−0.0076
F16	2008	0.1061	1.4358	−0.0087
F16	2009	0.1037	1.5615	−0.0095
F18	2010	0.0000	1.0000	0.0000
F18	2011	0.0727	1.2663	−0.0054
F18	2012	0.1187	1.1486	−0.0043
F18	2013	0.1218	1.2182	−0.0055

Following calibration, lighting from gas flares was masked out using polygon shapefiles of known gas flare locations available from the NOAA-NGDC^12^. Eliminating gas flares from the dataset is important because these do not reflect permanent population distribution.

1 $$y={C}_{0}+{C}_{1}x+{C}_{2}{x}^{2}$$

### Centroid location

The two algorithms used to locate nighttime lights centroids are described below. Each algorithm was implemented using the calibrated nighttime lights and GADM levels 0, 1, and 2 as inputs. For each GADM level an output point shapefile of the weighted nighttime light centre for each administrative area was created. Therefore, in total 132 output datasets were created, one for each of the 22 years, three GADM levels, and two different algorithms represented.

Barmore’s^10^ method for locating weighted centroids is an iterative method which uses an azimuthal-equidistant projection centred on the centroid itself to find the point which minimizes the sum of squared great circle distances from all weighted locations^19^. An azimuthal equidistant projection preserves distances and directions measured from the centre of the projection. This means that, when the weighted centroid is located at the centre of the projection itself, then distances between the centroid and each of the weighted points are true distances. Barmore’s process is described in detail in refs^19–21^.

First, a starting centroid was located using Eq. (2):

2 $${x}_{w}=\frac{{\sum }_{i=1}^{n}{w}_{i}{x}_{i}}{{\sum }_{i=1}^{n}{w}_{i}},\,{y}_{w}=\frac{{\sum }_{i=1}^{n}{w}_{i}{y}_{i}}{{\sum }_{i=1}^{n}{w}_{i}}$$

where *x*_*i*_ and *y*_*i*_ are the east-west and north-south coordinates of point *i*, respectively; *w*_*i*_ is the weight (DN) at point *i*; and *n* is the total number of points. The centre of each nighttime lights pixel was used as the weighting point *i*. For simplicity, a spherical datum with major and minor axes of 6371000 meters was used. The nighttime lights raster was projected into an azimuthal equidistant projection based on the same spherical datum and centred on the identified coordinates. A new centroid was then identified using Eq. (2) and the newly projected raster. The newly identified centre was then used to re-project the nighttime lights raster and once again calculate a new centre. This process was iterated until the distance between the newly identified centre and the centre used to establish the azimuthal equidistant projection was less than or equal to one meter.

Aboufadel and Austin’s^11^ method instead minimizes the sum of the squared straight-line distances from each of the weighted locations to the centre point^19^ by calculating the centre from a three-dimensional perspective. The centre is defined as the balance point of the population distribution as it resides on an idealized spherical representation of Earth’s surface. Aboufadel and Austin’s method is described in refs^11,19^.

The nighttime lights data were projected to the same spherical geographic coordinate system that was used for the Barmore method. The centre was then computed as the vector with coordinates given in Eq. (3):

3 $$\widehat{x}=\frac{{\sum }_{i}{w}_{i}{x}_{i}}{{\sum }_{i}{w}_{i}},\,\widehat{y}=\frac{{\sum }_{i}{w}_{i}{y}_{i}}{{\sum }_{i}{w}_{i}},\, {\hat{z}} =\frac{{\sum }_{i}{w}_{i}{z}_{i}}{{\sum }_{i}{w}_{i}}$$

where $\widehat{{\rm{x}}}$, $\widehat{{\rm{y}}}$ and $\widehat{{\rm{z}}}$ compose the centre vector; wi is the weight (DN) at the point i; and xi, yi, and zi compose the three-dimensional vector to point i, as given in Eq. (4):

4 $$\begin{array}{c}{x}_{i}={\rm{\cos }}{\lambda }_{i}{\rm{\cos }}{\varphi }_{i}\\ {y}_{i}={\rm{\sin }}{\lambda }_{i}{\rm{\cos }}{\varphi }_{i}\\ {z}_{i}={\rm{\sin }}{\varphi }_{{\boldsymbol{i}}}\end{array}$$

where ${\lambda }_{i}$ and ${\varphi }_{{\boldsymbol{i}}}$ are the longitude and latitude at point i, respectively. The longitude and latitude of the centre were then extracted using Eq. (5):

5 $$\widehat{\lambda }={{\rm{\tan }}}^{-1}\left(\frac{\widehat{y}}{\widehat{x}}\right),\,\,\widehat{\varphi }={{\rm{\sin }}}^{-1}\left(\frac{ {\hat{z}} }{\sqrt{{\widehat{x}}^{2}+{\widehat{y}}^{2}+{ {\hat{z}} }^{2}}}\right)$$

where $\widehat{{\rm{\lambda }}}$ and $\widehat{\varphi }$ are the longitude and latitude of the centre, respectively; and $\widehat{{\rm{x}}}$, $\widehat{{\rm{y}}}$ and $\widehat{{\rm{z}}}$ compose the centre vector.

Centres that did not fall within the corresponding administrative area were flagged, as were administrative areas that did not have a corresponding centre (see Data Records). The first case occurs when the shape of the administrative area is such that its weighted centre of nighttime lights does not fall within it. The second case occurs for one of two reasons. First, an administrative area will not have a corresponding centre if there are no lighted pixels found within it. Second, small administrative areas (smaller than or near to the nighttime lights pixel resolution) may be left without a centroid.

## Data Records

The centroid datasets described here can be freely and publicly accessed at the figshare web site^9^. The centroid datasets created were separated by algorithm, year, and GADM level. In total 132 different datasets were created each including a quality flag for each centroid point. Since each dataset is small in terms of storage they are grouped by algorithm and GADM level making the resulting data files 8 in total. In each shapefile (adm0, adm1, adm2) the field OUT_FLAG is used to store the flag value. In addition, there are three corresponding CSV-files, which report on unplaced centroids. The user can then decide how to proceed with the handling of unplaced centroids based on the specific user needs and application at hand. Possible options would be to force centroids to fall within a specific region based on some rule or just exclude them from analysis.

## Technical Validation

First, the consistency of the produced dataset was investigated. As mentioned in the previous section, sometimes it was not possible to define a population centre based on nighttime lights and sometimes they fall within another administrative unit. This is assessed and quantified. Second, distance between true population centres and derived centres are compared using Swedish census data.

### Centroid consistency

Centroids that did not fall within their corresponding administrative unit, or administrative areas that did not have a corresponding centroid were flagged. The flag values 0, 1 and 2 were used. 0- indicates point in region, 1- indicates point not in region and not on landmass, and 2- indicates point not in region but on land (i.e. it is in another region).

Table 3 is a summary for each year and reports on placed and unplaced centroids. There is no difference between the two methods at use. The number of placed centroids is increasing over the time period 1992–2013. This is not investigated further here but may be attributed to a combination of interacting factors such as increased economic development and possible improvements in satellite imagery over time. It is also noted that the trend is not linear but flickering between years. This is a pattern previously observed in countries with low electricity levels such as Burkina Faso where some villages and towns show unstable light patterns between years (submitted manuscript). It is possible that this could be an effect of processing heuristics and the thresholds used to pass as a lit area (lit 50% of the year).

**Table 3:** Expected and actual counts for each administrative level.

Year	Adm0 (N = 256)		Adm1 (N = 3609)		Adm2 (N = 46311)	
	Placed	Unplaced	Placed	Unplaced	Placed	Unplaced
1992	247	9	3255	354	37358	8953
1993	248	8	3382	227	40394	5917
1994	250	6	3415	194	41214	5097
1995	249	7	3411	198	41127	5184
1996	249	7	3420	189	41455	4856
1997	249	7	3429	180	41604	4707
1998	250	6	3470	139	42504	3807
1999	249	7	3459	150	42266	4045
2000	250	6	3482	127	42768	3543
2001	250	6	3477	132	42864	3447
2002	249	7	3458	151	42539	3772
2003	250	6	3485	124	42955	3356
2004	249	7	3491	118	43213	3098
2005	250	6	3490	119	43081	3230
2006	250	6	3486	123	43017	3294
2007	250	6	3484	125	43144	3167
2008	249	7	3473	136	42614	3697
2009	248	8	3444	165	41918	4393
2010	251	5	3516	93	43612	2699
2011	250	6	3509	100	43639	2672
2012	249	7	3488	121	43357	2954
2013	250	6	3486	123	43394	2917

### Distance between derived and true centroids

Next we compare the distance between each nighttime lights derived centroid to each true population centroid, for each administrative level and for both approaches (i.e. Barmore and Aboufadel & Austin). The true population centroid is derived from Swedish population data from Statistics Sweden, which is distributed on a 100 m by 100 m grid. Each grid cell contains the count of the number of people living there. This is based on data from the Swedish population register which has been geocoded to the location of the building of residence. All individuals that were registered as inhabitants in Statistics Sweden in 2007 and 2008 are included in the analysis and the data were aggregated to 500 m spatial resolution due to confidentiality reasons. The data was obtained in an RT90 25 gon väst projection; however, the distance between true and nighttime lights centroids was calculated in a SWEREF99 projection since this is a more current projection for Sweden.

Population data from Sweden was used as a benchmark partly because of outstanding annual population data with a very high spatial resolution and partly because Sweden varies greatly in population distribution and shape of administrative units. Therefore, it can be representative for many parts of the world. We do, however, recognize that the relationship between the true and derived centroids which we test may be stronger in developed countries such as Sweden than in developing regions. It has been shown that agricultural based economies in developing regions emit low levels of light which can be difficult to separate from image noise^20,22,23^ and therefore produce a weaker link to population distribution. However, we expect that while the link between the true and nighttime lights centroid may be weaker in developing countries with lower light levels, there is still an added benefit of such estimating the nighttime lights centroid due to the fact that other, better population data may not exist or be readily available.

**Fig. 1:**
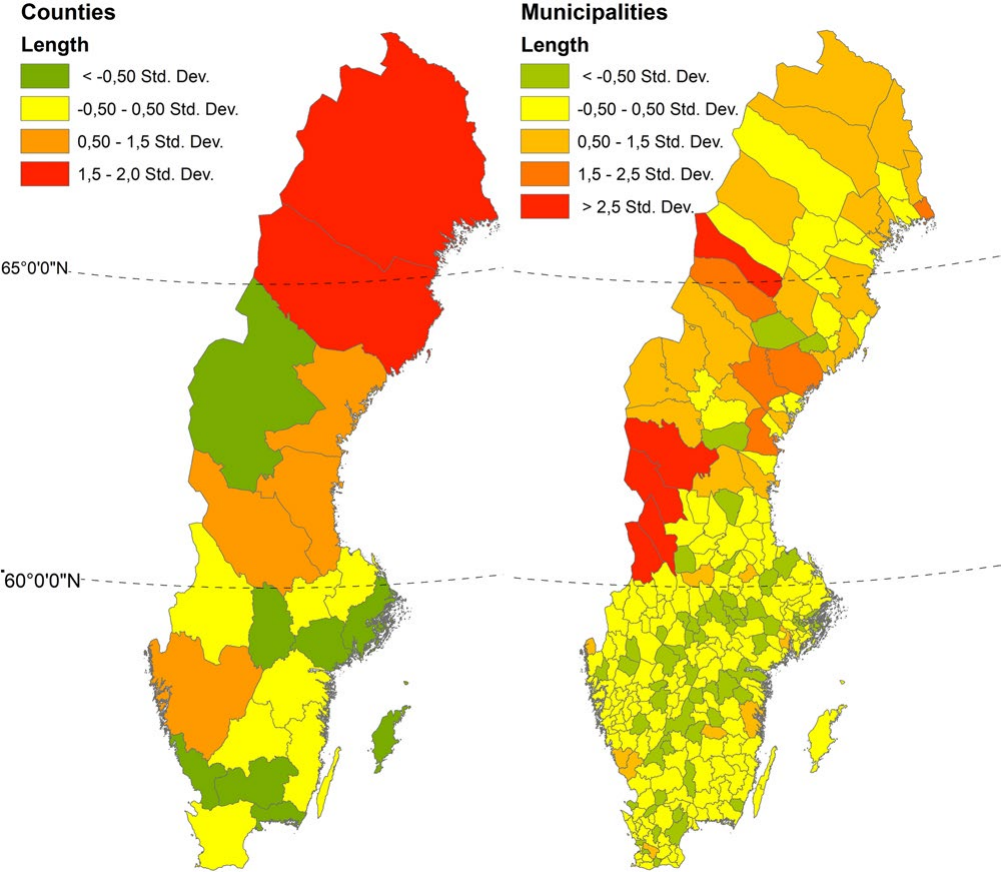
Comparison of distance between NTL centroids and true centroids for the year 2008 in Sweden per administrative level. Numbers shown are deviations from the mean distance error between derived and true centroids. Errors tend to be larger in the northern half of Sweden.

Figure 1 describes the distribution of distance error between true and derived centroids. The influence of the area, shape and population density of the regions were previously compared to distance between centroids^6^. The only significant correlation found was between distance between centroids and area of region (R^2^ = 0.1213). This means that the larger the administrative region, the larger the distance between centroids. This error is most pronounced in the northern parts of Sweden, which contains larger regions as compared to the south. The population density follows much the same pattern, with low-density regions in the North and high-density regions in the South.

The discrepancies described can also possibly be attributed to the inherent properties of the nighttime light satellite sensor. Known issues are saturation in brightly lit areas and overestimation of lit area (blooming) due to sensor sensibility^2^. In related works, areas above 65°N are regularly masked out due to inconsistencies based on the fact that the Sun sets late and high amount of aurora activity^6^. This issue affects 7 countries and 0.036% of the global population. Care should be taken for regions above 60°N as these effects increase with latitude.

### Comparison between centroid estimation methods

**Fig. 2:**
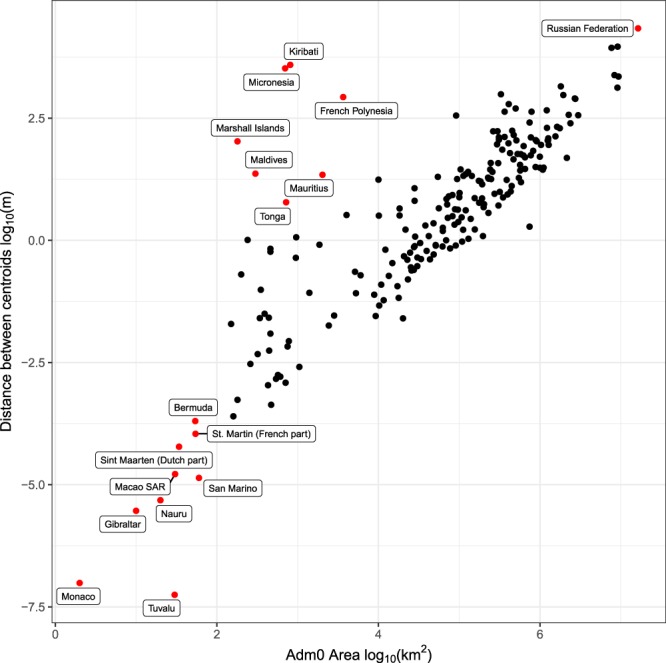
Comparison between methods for each country. Numbers shown are distance between derived centroids (y-axis) and size of administrative area (x-axis). The R-code to generate this figure is found in the repository.

A comparison between the methods was done for each country and year 2008 by relating the differences in the centroids with the area of the administrative unit (Fig. 2). The distance between the centroid locations increases with area. However, some of the smaller countries show a higher than expected distance which is because they are island nations which cover a much larger total extent but have a smaller total area. From this we can conclude that the selection of method is more important when the area of the interested regions is large or when the regions cover a large extent.

## Data Availability

The resulting centroid datasets were created using R version 3.0.2 (R: A Language and Environment for Statistical Computing, https://www.r-project.org/) and the rgdal and maptools packages (https://CRAN.R-project.org/package=rgdal, https://CRAN.R-project.org/package=maptools). The scripts to generate the data and the data can be found on.
